# Novel serum protein biomarker panel revealed by mass spectrometry and its prognostic value in breast cancer

**DOI:** 10.1186/bcr3676

**Published:** 2014-06-16

**Authors:** Liping Chung, Katrina Moore, Leo Phillips, Frances M Boyle, Deborah J Marsh, Robert C Baxter

**Affiliations:** 1 Hormones and Cancer Division, Kolling Institute of Medical Research, University of Sydney, Royal North Shore Hospital, Reserve Road, St Leonards, NSW 2065, Australia; 2 Department of Breast Endocrine Surgery, Royal North Shore Hospital, Reserve Road, St Leonards, NSW 2065, Australia; 3 Patricia Ritchie Centre for Cancer Care and Research, Mater Hospital, 13 Gillies Street, Crows Nest, NSW 2065, Australia

## Abstract

**Introduction:**

Serum profiling using proteomic techniques has great potential to detect biomarkers that might improve diagnosis and predict outcome for breast cancer patients (BC). This study used surface-enhanced laser desorption/ionization time-of-flight (SELDI-TOF) mass spectrometry (MS) to identify differentially expressed proteins in sera from BC and healthy volunteers (HV), with the goal of developing a new prognostic biomarker panel.

**Methods:**

Training set serum samples from 99 BC and 51 HV subjects were applied to four adsorptive chip surfaces (anion-exchange, cation-exchange, hydrophobic, and metal affinity) and analyzed by time-of-flight MS. For validation, 100 independent BC serum samples and 70 HV samples were analyzed similarly. Cluster analysis of protein spectra was performed to identify protein patterns related to BC and HV groups. Univariate and multivariate statistical analyses were used to develop a protein panel to distinguish breast cancer sera from healthy sera, and its prognostic potential was evaluated.

**Results:**

From 51 protein peaks that were significantly up- or downregulated in BC patients by univariate analysis, binary logistic regression yielded five protein peaks that together classified BC and HV with a receiver operating characteristic (ROC) area-under-the-curve value of 0.961. Validation on an independent patient cohort confirmed the five-protein parameter (ROC value 0.939). The five-protein parameter showed positive association with large tumor size (*P* = 0.018) and lymph node involvement (*P* = 0.016). By matrix-assisted laser desorption/ionization time-of-flight (MALDI-TOF) MS, immunoprecipitation and western blotting the proteins were identified as a fragment of apolipoprotein H (ApoH), ApoCI, complement C3a, transthyretin, and ApoAI. Kaplan-Meier analysis on 181 subjects after median follow-up of >5 years demonstrated that the panel significantly predicted disease-free survival (*P* = 0.005), its efficacy apparently greater in women with estrogen receptor (ER)-negative tumors (*n* = 50, *P* = 0.003) compared to ER-positive (*n* = 131, *P* = 0.161), although the influence of ER status needs to be confirmed after longer follow-up.

**Conclusions:**

Protein mass profiling by MS has revealed five serum proteins which, in combination, can distinguish between serum from women with breast cancer and healthy control subjects with high sensitivity and specificity. The five-protein panel significantly predicts recurrence-free survival in women with ER-negative tumors and may have value in the management of these patients.

## Introduction

The diagnosis of breast cancer relies on an integrated approach using clinical and physical examinations, imaging mammography and ultrasound, and histopathology. Although serum biomarkers have not yet played a major role in breast cancer diagnostic or prognostic practice [1,2], an effective biomarker panel in an easily accessible biological fluid would be a valuable and minimally invasive adjunct to other clinical and pathological approaches [3]. As whole blood provides a dynamic representation of physiological and pathological status, serum or plasma represents the most extensively studied biological matrix for cancer biomarkers [4]. Therefore, analysis of the serum or plasma proteome may be an important step to achieve accurate diagnosis or prognosis.

For breast cancer biomarker discovery, proteins and peptides have been identified in breast cancer cell lines [5-7], nipple aspirate fluid [8,9], and normal, benign, premalignant, and malignant breast tissue [10-13], in addition to serum and plasma [1,4,14]. Numerous proteomics-based studies of serum and plasma have reported discriminatory peptide/protein ion peaks, either as identified proteins or on the basis of their mass/charge (*m/z*) values, for breast cancer diagnosis or prognosis. However, not all have reported protein identities for the discriminatory ion peaks.

This study used surface-enhanced laser desorption/ionization time-of-flight (SELDI-TOF) mass spectrometry (MS) protein chip technology to discover a unique combination of serum biomarkers for breast cancer and confirm them in an independent sample set. The markers were identified by matrix-assisted laser desorption/ionization time-of-flight (MALDI-TOF)/TOF MS and verified immunologically. We also investigated the association between this serum protein panel and patient outcome to determine its potential prognostic utility.

## Methods

### Serum samples

The study involved a total of 320 human serum specimens and was approved by the Human Research Ethics Committee of the Northern Sydney Local Heath District, Sydney, Australia. The training set samples from patients diagnosed with breast cancer (BC, n = 99) and control samples from healthy volunteers (HV, n = 51) were obtained from the Kolling Institute Breast Tumour Bank, at the Royal North Shore Hospital, Sydney, Australia. The validation set consisted of 100 independent BC serum samples from the Australian Breast Cancer Tissue Bank, Sydney, Australia and 70 HV samples. Sample sizes were estimated to allow the detection of a difference of at least 25% in a measured parameter between sample groups at the 5% significance level (α = 0.05) with a statistical power of at least 0.8, assuming group coefficients of variation of 50% (σ = 0.5). All patients whose tumor samples (or healthy tissue samples) are deposited into either of the two tissue banks used in this project had given prior written informed consent to the banking of their tissue and its use in any future research projects. Therefore additional patient consent was not required for this specific project. The median ages of patients included in the training and validation sets were 59 (range 28 to 92) and 58 (31 to 86), respectively. For HV control groups, serum samples were age-matched to BC samples within five-year age brackets. All sera were stored at -80 ºC until analyzed by SELDI-TOF MS.

### Preparation of serum sample and protein chips for SELDI-TOF MS

All serum samples were initially denatured in buffer containing 8 M urea, 1% CHAPS (3-[(3-cholamidopropyl) dimethylammonio]-1-propanesulfate) and analyzed by TOF MS on SELDI protein chip arrays (Bio-Rad, Hercules, CA, USA) as previously described [15]. Four chip types with different adsorptive surfaces were used: Q10 (strong anion-exchange), Cu^2+^-IMAC30 (immobilized metal affinity capture), CM10 (weak cation-exchange), and H50 (hydrophobic). The four chip types were pre-equilibrated twice for 5 min with 5 μl of binding buffer (50 mM Tris-HCl pH 8.0 for Q10; phosphate-buffered saline (PBS) pH 7.2 for IMAC30; 50 mM sodium acetate pH 6.0 for CM10; 10% acetonitrile (ACN) containing 0.1% trifluoroacetic acid (TFA) for H50). Denatured serum protein samples were diluted 1:5 with the respective binding buffers and 5 μl of each diluted sample was pipetted onto the chips. All samples were analyzed in duplicate. Chips were then incubated with shaking for 90 min at room temperature (settings: form 20, amplitude 4) on a MicroMix 5 (EURO/DPC Instrument Systems, Flanders, NJ, USA). After washing twice with the binding buffer, each spot was treated with 2 × 1 μl of 50% sinapic acid (Sigma-Aldrich, St Louis, MO, USA) in 50% ACN, 0.5% TFA and air dried.

### MS serum protein profiling and data analysis

All mass spectra were obtained in the *m/z* range of 2,500 to 70,000 with the ProteinChip SELDI System Enterprise Edition (Bio-Rad). Spectra were averaged from 583 laser shots evenly distributed across each spot. Mean values from duplicate spectra for each sample were used in all subsequent analyses. The *m/z* value for each peak was determined using external calibration with protein standards: bovine insulin (5,734.51 Da), equine cytochrome *c* (12,361.96 Da), equine apomyoglobulin (16,952.27 Da) and bovine carbonic anhydrase (29,023.70 Da) from Sigma-Aldrich. After calibration, spectra were baseline-subtracted and normalized using the total ion current between 2,500 and 30,000 *m/z*. Of the original 320 samples, 19 were excluded when their mass spectra did not meet the normalization criteria. A total of 602 spectra were subjected to full analysis (301 samples: BC = 187 and HV = 114, in duplicate) on each of four chip types (total = 2,408 spectra).

Clustering analysis of protein peaks (ProteinChip Data Manager version 4.1, Bio-Rad) was performed to identify protein patterns related to BC and HV groups. Data analysis across all four protein chip types was achieved using univariate analysis of individual peaks by Mann-Whitney *U* test (IBM SPSS version 20.0, IBM Corp., Armonk, NY, USA). For initial discovery, biomarker panels were developed on the training data set of 99 BC and 51 HV samples. All protein peaks that significantly discriminated BC from HV at *P* <0.005 were then subjected to multivariate analysis using forward and reverse binary logistic regression (SPSS) to develop the training model. The discriminatory power of each putative serum biomarker was further described using receiver operating characteristic (ROC) area-under-the-curve (AUC) analysis [10,16]. External validation was also carried out using an independent set of 100 BC and 70 HV serum samples aged-matched within five-year age brackets.

### Protein peak identification

Immunological validation of protein biomarkers and protein peak identification was achieved by immunodepletion using Protein G Dynabeads (Life Technologies Corp., Carlsbad, CA, USA). For complement C3a des-arginine anaphylatoxin (C3a-desArg), 1.5 mg of Protein G beads was incubated with 5 μg of anti-C3a/C3a desArg mouse monoclonal antibody (Abcam, Cambridge, UK) and incubated for 30 min at room temperature with rotation. After washing with 200 μl of PBS containing 0.02% Tween 20 to remove free antibody, the immobilized antibody was added to 50 μl of diluted serum samples and incubated for 2 h at 4 ºC with rotation. The captured protein-antibody complex was washed twice with 200 μl of PBS and the bound protein eluted at room temperature in 20 μl of 0.1 M glycine, pH 3.0. The starting material, immunodepleted samples and the eluted proteins were monitored by SELDI-TOF MS on normal-phase NP20 chips (Bio-Rad). For apolipoprotein CI (ApoCI) and transthyretin (TTR) a similar procedure, using rabbit anti-ApoCI polyclonal antibody (Abcam) and anti-prealbumin monoclonal antibody (Abcam) respectively, was followed.

### Immunological confirmation of serum protein markers by western blotting

Three putative protein markers were also examined by western blotting. Human sera from BC (*n* = 4) and HV (*n* = 4) were separated by 4 to 12% SDS-PAGE (Invitrogen, Carlsbad, CA, USA) and transferred to polyvinylidene difluoride membrane (Bio-Rad). Membranes were blocked for 1 h at room temperature with 5% skim milk. Western blotting was conducted using primary antibodies against C3a/C3a desArg (mouse monoclonal, Abcam) or ApoCI (rabbit polyclonal, Abcam) at 1:1000 dilution and TTR (mouse monoclonal antibody to human prealbumin, Abcam) at 1:2000 in 5% skim milk. Secondary antibodies, peroxidase-linked anti-mouse immunoglobulin G (IgG) (1:2000) or anti-rabbit IgG (1:2000), respectively, were added for 1 h at room temperature and protein bands were visualized by enhanced chemiluminescence using Amersham ECL Prime Western Blotting Detection Reagent (GE Healthcare Life Sciences, Little Chalfont, UK). Western blot data were imaged using the LAS 3000 imaging system (Fujifilm, Stamford, CT, USA) and the images were analyzed with MultiGauge version 3.0 software (Fujifilm). Correlations between densitometric analysis by western blotting and SELDI-MS peak intensities were also examined.

### Protein identification by MALDI-TOF/TOF MS

Human sera were fractionated on a weak cation-exchange HiTrap FF column (GE Healthcare) with a linear gradient from 0 to 600 mM NaCl in 25 mM Na acetate pH 6.0 using an ÄKTA Purifier system (GE Healthcare). Fractionated proteins were monitored by SELDI-TOF MS on NP20 chips. Fractions containing a 3.8 kDa putative biomarker were further purified using reverse-phase liquid chromatography (RP-LC) on a 250 × 4.6 mm Jupiter 5 μm 300-Å C18 column (Phenomenex, Lane Cove, Australia), eluted with a 30-min linear gradient from 15 to 60% ACN in 0.1% TFA at 1.5 ml/min. After freeze drying the fraction containing the protein of interest, it was reconstituted in 15% ACN, 0.1% TFA and analyzed using MALDI-TOF peptide mass fingerprinting (PMF) and MS/MS on a Bruker UltrafleXtreme MALDI-TOF/TOF MS (Bruker Daltonics, Bremen, Germany), using an MTP AnchorChip target (Bruker Daltonics) and α-cyano-4-hydroxycinnamic acid as matrix.

To identify the protein peak at 28.2 kDa, human sera were fractionated by anion-exchange chromatography using Q ceramic HyperD F sorbent (BioRad) by means of stepwise pH elution from pH 9 to pH 4 as previously described [17]. Fractionated proteins were monitored by SELDI-TOF MS on NP20 chips. Final identification was achieved after liquid chromatography (Ultimate 3000 nanoLC, Thermo Fisher Scientific, Waltham, MA, USA) on an Acclaim PepMap RSLC C18 2 μm, 100 Å, nanoViper guard (75 μm × 20 mm) and analytical (75 μm × 150 mm) column (Thermo Fisher Scientific), using a 2 to 79% ACN/0.05%TFA gradient at 300 nl/min. Fractions were spotted onto an MTP AnchorChip target (Bruker), and analyzed by MS/MS using the UltrafleXtreme MALDI-TOF/TOF MS (Bruker).

### Statistical analysis

Univariate analysis by the Mann-Whitney *U* test (SPSS Inc., Chicago, IL, USA) was used to distinguish sera from patients with breast cancer from healthy controls. Further multivariate analysis by binary logistic regression was also achieved by SPSS. The correlation between the levels of the five serum markers, individually and in combination, with tumor pathologic variables (histological grade, tumor size, lymph node involvement, estrogen receptor (ER) and progesterone receptor (PR) status and human epidermal growth factor receptor 2 (HER2) overexpression) were investigated by multiple linear regression (SPSS). We defined the median of combined peak intensity for all group patients as the cutoff value for the survival data analysis. Disease-free survival analyses were estimated using the Kaplan-Meier method and the model differences in survival time were tested using the log-rank test.

## Results

### Patient characteristics

Sera analyzed in this study formed two sample sets, the training and validation groups. Of 320 serum samples (BC = 199, HV = 121) selected for both the training and validation sets, 187 BC and 114 HV were fully analyzed, 19 samples (12 BC, 7 HV) being excluded because their mass spectra did not meet the normalization criteria. The pathological characteristics of the tumors including histological type and grade, size, hormone receptor (ER and PR), HER2 status as well as lymph node status are presented in Table 1. More than 90% of cancer patients had an invasive tumor and more than half of these were of high histological grade. Clinical management of all patients was based on standard guidelines [18] and treatment involved radiotherapy, chemotherapy, hormone therapy, and trastuzumab in patients with HER2-positive tumors. Median follow-up for ER-positive patients (n = 131) was 5.10 years (range: 0.16 to 9.50) and for ER-negative patients (n = 50), 5.05 years (range: 0.12 to 9.42).

**Table 1:** Patient characteristics

	**Training set**	**Validation set**
Number of patients	92	95
Age median	59	58
**Histologic type**		
IDC	75	54
ILC	8	8
Other	9	33
**Histologic grade**		
G1	8	14
G2	33	31
G3	47	49
Missing	4	1
**Tumor size**		
T ≤2 cm	34	65
T >2 cm	55	30
Missing	3	
**Estrogen receptor**		
Positive	58	73
Negative	28	22
Missing	6	
**Progesterone receptor**		
Positive	48	73
Negative	41	22
Missing	3	
**HER2 overexpression**		
Positive	17	21
Negative	60	73
Missing	15	1
**Lymph node involvement**		
Positive	43	36
Negative	41	37
Missing	8	22

HER2, human epidermal growth factor receptor 2; IDC, invasive ductal carcinoma; ILC, invasive lobular carcinoma.

### Establishment of a putative biomarker panel

A total of 320 serum samples were used in this study generating 2,560 mass spectra. After normalization, spectra from 19 subjects were excluded from the data analysis, leaving 2,408 spectra (4 chip surfaces, 187 BC and 114 HV, in duplicate) available for full data analysis. From the 92 BC and 46 HV serum samples included in the training set, 57 common peaks from H50 chips, 62 from IMAC30, 70 from CM10, and 57 from Q10, were determined by clustering analysis using expression differential mapping (Bio-Rad). Of these, a total of 51 peaks - 14 from H50 (Figure S1A in Additional file 1), 12 from IMAC30 (Figure S1B in Additional file 1), 13 from CM10 (Figure S1C in Additional file 1), and 12 from Q10 (Figure S1D in Additional file 1) - were differentially expressed as determined by Mann-Whitney *U* test (*P* <0.005, Table S1 in Additional file 2). When tested for their ability to discriminate between BC and HV samples, these peaks gave individual ROC-AUC values ranging from 0.65 to 0.84 (Table S1 in Additional file 2). Note that for downregulated proteins, ROC-AUC values are expressed as (1-value). The 51 significant peaks were then tested in a forward and reverse binary logistic regression analysis with 10-fold cross-validation.

**Figure 1:**
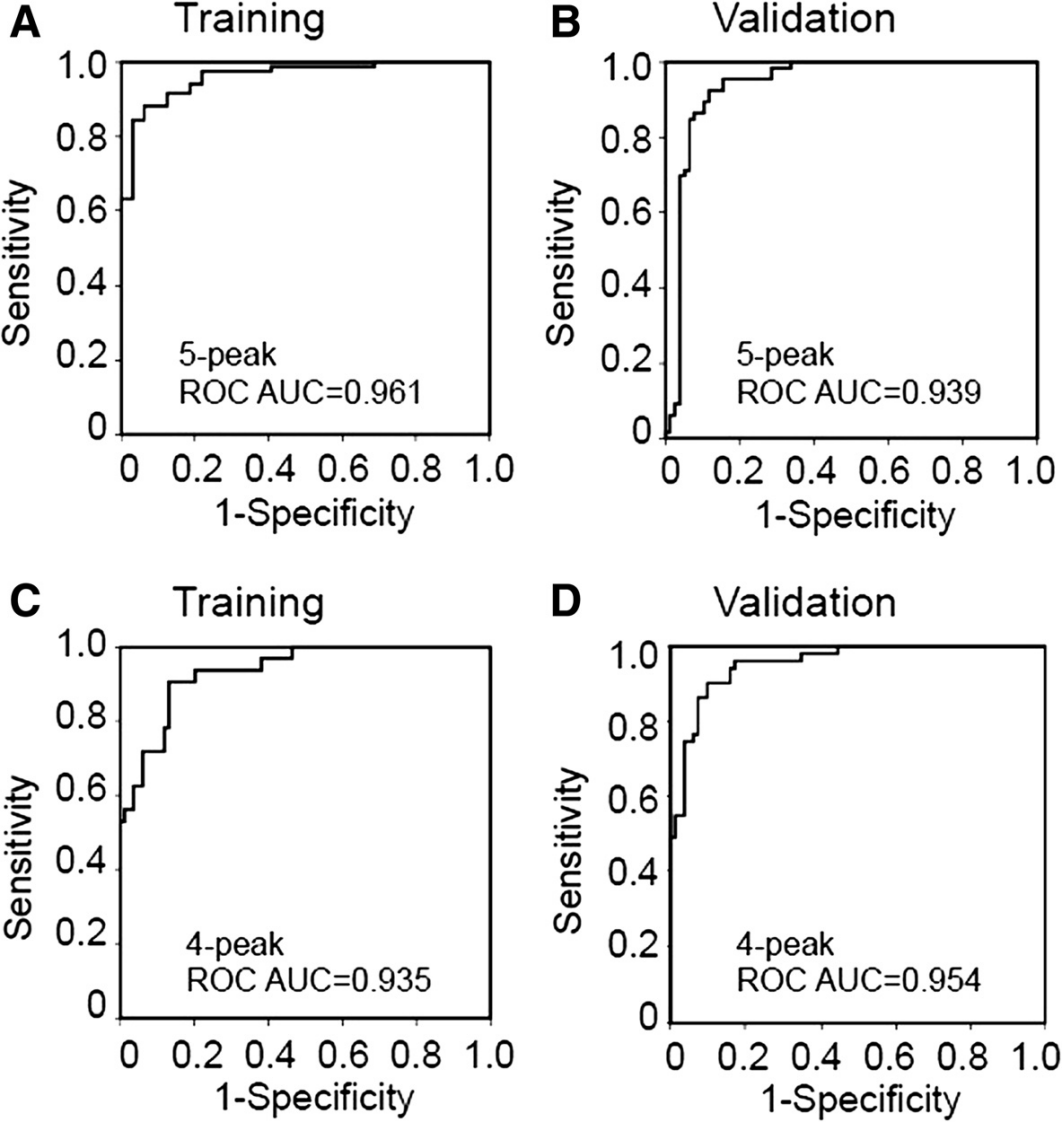
**Receiver operating characteristic (ROC) curve analysis comparing the performance of five-protein and four-protein panels on training and validation data sets.** When used to distinguish serum samples from breast cancer patients (BC) from those from the healthy volunteer (HV) group, the average ROC area under the curve (AUC) was 0.961 for the five-protein parameter in the training set **(A)** and 0.939 in the independent validation set **(B)**. For the four-protein parameter, the average value of ROC-AUC was 0.935 in the training set **(C)** and 0.954 in the validation set **(D)**.

**Figure 2:**
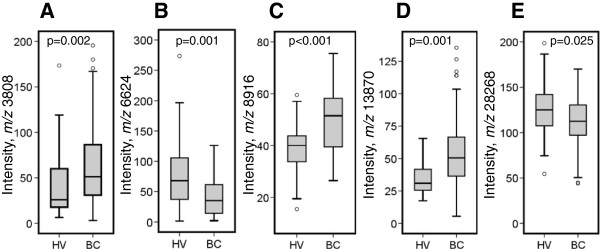
**Relative abundance of the serum proteins used to develop the five-protein parameter.** Significantly different peak intensities observed between breast cancer patient (BC) and healthy volunteer (HV) samples on the combined training and validation sets: **(A)***m/z* 3808 on CM10 weak cation-exchange surface; **(B)***m/z* 6624 on H50 hydrophobic surface; **(C)***m/z* 8916 on Q10 strong anion exchange surface; **(D)***m/z* 13870 on IMAC30 immobilized metal affinity surface; **(E)***m/z* 28268 on Q10 strong anion exchange surface. Data were analyzed by the Mann-Whitney *U* test.

The final training model gave an average ROC-AUC value of 0.961 (Figure 1A, Table 2) for a combination of five peaks (*m/z* 3808, *m/z* 6624, *m/z* 8916, *m/z* 13870, and *m/z* 28268). By univariate analysis, the intensities of peaks with *m/z* 3808, *m/z* 8916 and *m/z* 13870 were significantly higher in BC serum than in HV serum (*P* = 0.002, *P* <0.001, and *P* = 0.001, respectively), while the intensities of peaks with *m/z* 6624 and *m/z* 28268 were significantly lower in BC serum than in HV (*P* = 0.001 and *P* = 0.025) (Figure 2).

**Table 2:** Diagnostic performance of five-protein and four-protein classification models

**Model**	**Cancer patients**	**Healthy volunteers**	**Sensitivity (%)**	**Specificity (%)**	**Overall accuracy (%)**	**ROC-AUC**
Five-protein panel						
Training set	92*	46*	97.6	87.1	93.1	0.961
Validation set	95*	68*	86.8	92.4	89.4	0.939
Four-protein panel						
Training set	92*	46*	90.5	79.9	85.3	0.935
Validation set	95*	68*	92.6	82.4	88.6	0.954

*included mass spectrometry (MS) spectra after normalization. ROC-AUC, receiver operating characteristic-area under the curve.

For validation, the five putative biomarkers were tested using an independent data set of mass spectra derived from 100 BC serum samples and 70 age-matched HVs, of which 1,304 spectra (95 BC and 68 HV in duplicate, 4 chip surfaces) could be analyzed after normalization. Testing the five-protein panel derived from the training set on the independent sample set gave an average ROC-AUC value of 0.939 (Figure 1B). The sensitivity and specificity of the combined five-protein panel were 86.6% and 92.4% respectively, and overall accuracy was 89.4% (Table 2).

In an attempt to simplify the model, we also tested a four-protein panel (*m/z* 6624, *m/z* 8916, *m/z* 13870, and *m/z* 28268) that omitted the *m/z* 3808 peak (for which we had no immunological confirmation), to compare with the original training model of five proteins. By multivariate analysis, the simplified model applied to the training set gave an average of ROC-AUC value of 0.935 (Figure 1C). The sensitivity and specificity of the combined four-protein panel were correspondingly reduced to 90.5% and 79.9%, respectively (Table 2). However, testing the four-protein panel on the validation set gave an average ROC-AUC value of 0.954 with sensitivity and specificity of 92.6% and 82.4% (Figure 1D, Table 2). Therefore the simplified model consisting of four proteins, omitting the *m/z* 3808 peak, also has considerable discriminatory power.

### Identities of protein peaks confirmed by immunodepletion and western blotting

**Figure 3:**
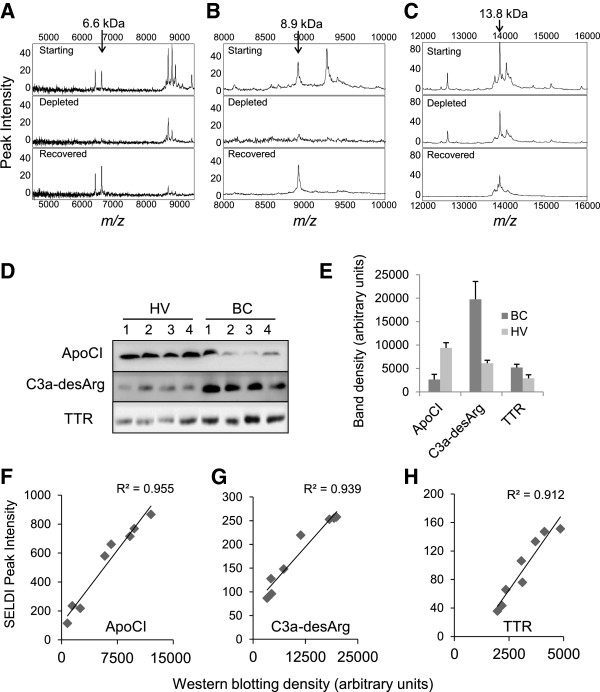
**Identification of serum proteins by immunodepletion and western blotting.** Proteins representing peaks at *m/z* 6624, *m/z* 8916 and *m/z* 13870 were immunodepleted from serum extracts using monoclonal antibodies against **(A)** apolipoprotein CI (ApoCI), **(B)** C3a/ C3a des-arginine anaphylatoxin (C3a-desArg) or **(C)** transthyretin. The starting material (Starting, upper panel), the immunodepleted sample (Depleted, middle panel) and the eluted fraction (Recovered, lower panel) were analyzed on NP20 arrays by surface-enhanced laser desorption/ionization time-of-flight (SELDI-TOF) mass spectrometry (MS). **(D)** Western blotting for ApoCI, C3a/C3a-desArg and transthyretin were performed on four healthy volunteer (HV) samples and four breast cancer patient (BC) samples. **(E)** Mean band densities (+standard deviation (SD)) derived from the blots shown in panel D (n = 4 for all groups). **(F-H)** Association between SELDI peak intensities and western blotting band densities for individual HV and BC serum samples (n = 8), indicating strong correlations for **(F)** ApoCI, **(G)** C3a-desArg and **(H)** transthyretin.

Serum protein peaks at *m/z* values similar to 6,624 and 8,916 have been previously reported in the literature from other MS-based studies, identified as ApoCI and C3a-desArg, respectively [19-21]. To confirm these identities for our peaks of *m/z* 6624 and 8916, proteins were enriched by immunoprecipitation from serum extracts using immobilized anti-ApoCI or C3a/C3a desArg antibodies. Both the immune-depleted serum and the immunoprecipitated proteins were analyzed by SELDI-TOF MS. The peak at *m/z* 6624 (Figure 3A) was fully depleted by ApoCI antibody and recovered in the immunoprecipitate, and the peak at *m/z* 8916 (Figure 3B) was approximately 80% depleted by the C3a/C3a-desArg antibody and also recovered in the immunoprecipitate, confirming the identities of these protein peaks. Interestingly, a peak at *m/z* 6428 co-precipitated with full-length ApoCI (*m/z* 6624), suggesting that *m/z* 6428 is related to ApoCI. For the peak at *m/z* 13870, assumed to be transthyretin (TTR, also known as prealbumin), immunoprecipitation with immobilized anti-human prealbumin antibody partially depleted the serum extract, and again the depleted protein was recovered in the precipitate (Figure 3C).

Figure 3D compares the levels of ApoCI, C3a/C3a-desArg and TTR by western blotting between four BC and four HV serum samples, with quantitation of band densities shown in Figure 3E, correlations between SELDI peak intensity and densitometric analysis of western blotting are shown in Figure 3F, G and H for the three proteins, indicating strong positive relationships between MS peak intensity for peaks identified as ApoCI (*m/z* 6624), C3a-desArg (*m/z* 8916), and TTR (*m/z* 13870), and their quantitation by immunoblot.

### Protein peaks identified by MALDI-TOF MS/MS

**Figure 4:**
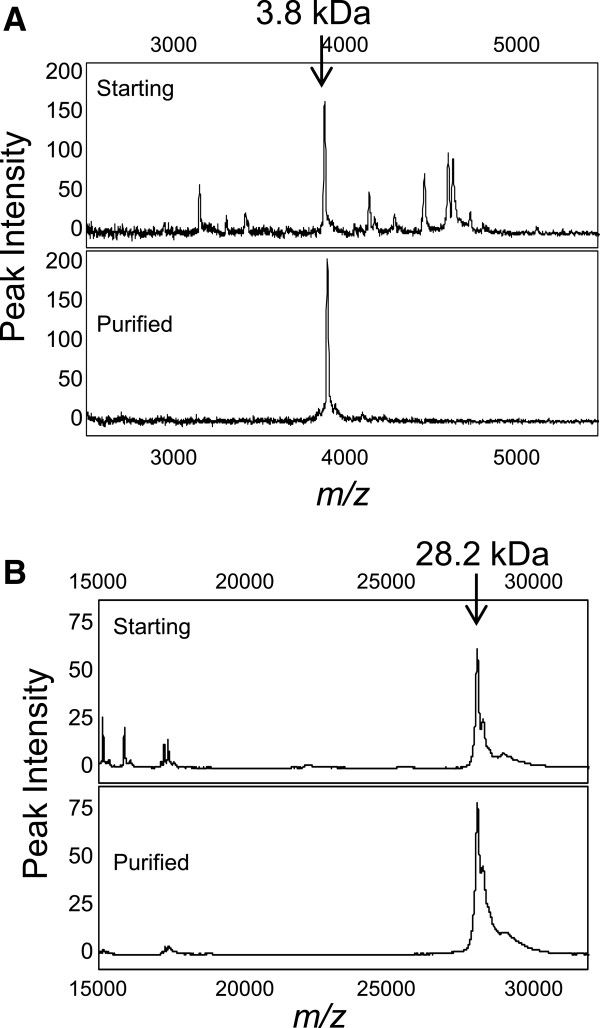
**Surface-enhanced laser desorption/ionization (SELDI) profiles before and after protein purification. (A)***m/z* 3808: the starting material (upper panel) and the purified fraction (lower panel) after weak cation chromatography and reverse-phase high-performance liquid chromatography (RP-HPLC) were analyzed on NP20 arrays. **(B)***m/z* 28268: the starting material (upper panel), and the purified fraction (lower panel) after strong anion-exchange and RP-HPLC were analyzed on NP20 arrays.

To identify the 3.8 kDa protein, it was purified using cation-exchange and reverse-phase liquid chromatography (RP-LC) and analyzed by MALDI-TOF MS. Figure 4A shows a SELDI-TOF spectrum of the 3.8 kDa peptide from whole serum (upper panel) and the peptide after purification by two chromatographic procedures using HiTrap CM FF followed by RP-LC (lower panel). MALDI-TOF/TOF MS/MS indicated a fragment of apolipoprotein H (ApoH or beta2 glycoprotein 1, P02749) based on a single tryptic peptide of *m/z* 1446 (Table S2 in Additional file 3), or 38% of the mass of the peptide observed by SELDI-TOF MS (*m/z* 3808). Since full-length human ApoH is 38.3 kDa, the *m/z* 3808 biomarker peptide represents a small fragment of ApoH. It was not possible to analyze this ApoH fragment immunologically as no antibody specific for the identified sequence is currently available.

The 28.2 kDa protein was purified by anion exchange and RP-HPLC and the fractions containing this protein were analyzed by SELDI-TOF MS using NP20 Protein Chip arrays. Figure 4B shows SELDI mass spectra for this peak before and after two chromatographic procedures. MALDI-TOF/TOF MS identified full-length mature human ApoAI (P02647) with total sequence coverage more than 90%, as indicated by the identified peptides shown in Table S2 in Additional file 3. Western blotting for ApoAI showed no significant difference in band intensity between four BC and four HV samples (data not shown).

The immunoprecipitated protein of *m/z* 13870 was also confirmed as TTR by MALDI-TOF/TOF MS/MS after tryptic digestion (Table S2 in Additional file 3) giving three peptide sequences mainly at the C-terminus of TTR. By MALDI-TOF MS analysis the main peak (undigested) appeared at *m/z* 13756 (not shown), smaller than the size of 13,870 determined by SELDI-TOF MS.

### Prognostic value of the serum biomarker panel

The median follow-up periods for patients in the training and validation sets were 6.1 (0.16 to 9.50) years and 4.8 (0.12 to 6.58) years, respectively. Of 181 patients with follow-up data available, 86 were in the training set and 95 in the validation set. During the follow-up period, recurrence of breast cancer occurred in 32 patients (17.7%) and death occurred in 33 patients including 22 (12.2%) who died due to breast cancer and distant metastases, and 11 (6.1%) who died from other causes. Disease-free survival was calculated from the time of surgery to the time that death or distant metastasis was recorded, or the date of the last follow-up for all censored patients. Survival estimates were analyzed by the Kaplan-Meier method and compared by the log-rank test. We defined the median value of the combined serum values for the five- or four-peak panels (derived by SPSS) for all breast cancer patients as the cutoff value for each survival data analysis and compared the outcomes produced by the two models (five-peak panel vs. four-peak panel).

**Figure 5:**
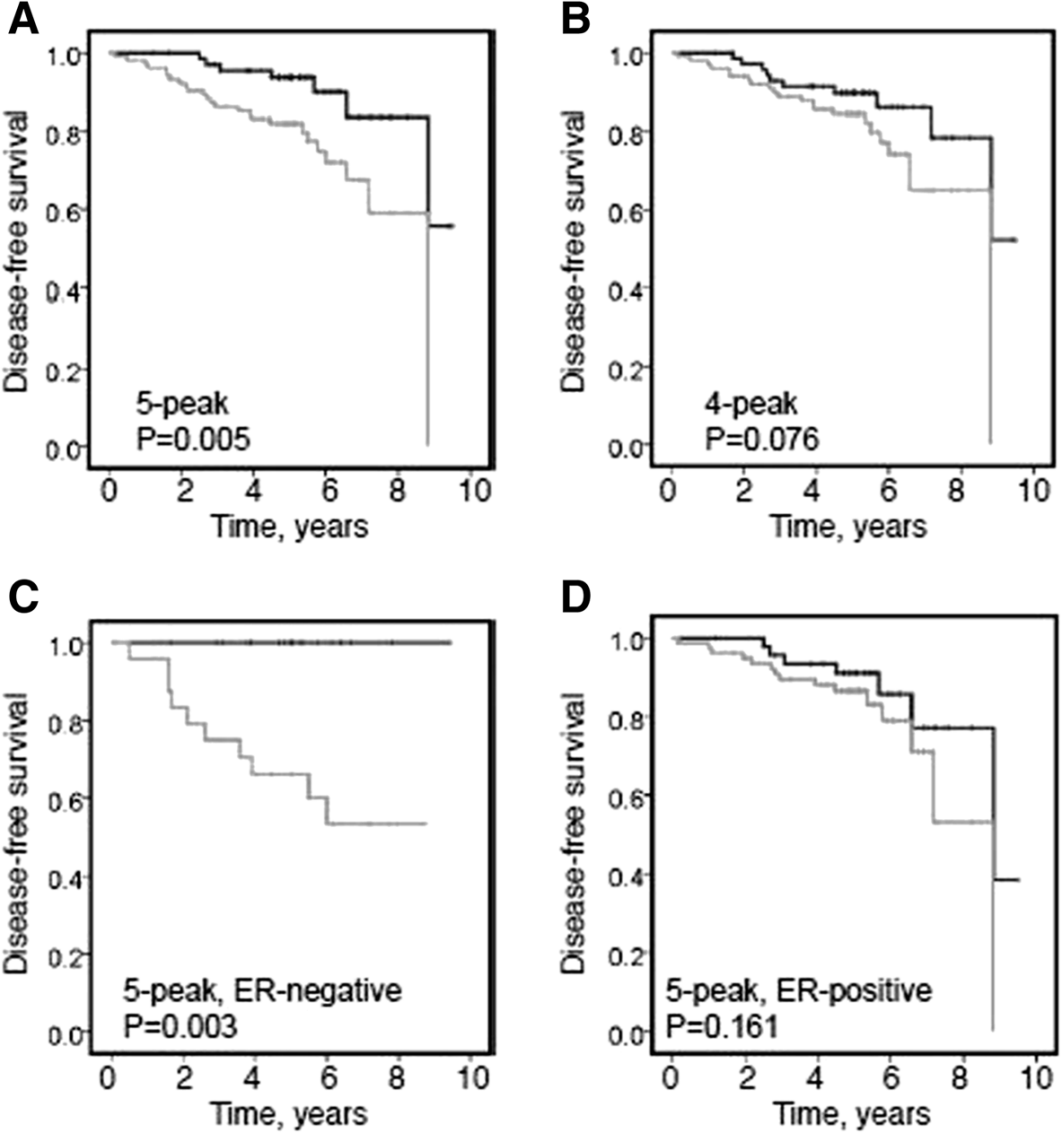
**Kaplan-Meier survival analyses. (A)** Patients with values of the five-protein parameter greater than the median (gray line) showed significantly worse disease-free survival than those with values below the median (black line) **(B)** Similar data for the four-protein parameter, omitting the *m/z* 3808 peak (apolipoprotein H (ApoH) fragment). **(C)** In women with estrogen receptor (ER)-negative tumors, values greater than the median for the five-protein parameter (gray line) were associated with significantly worse survival than values below the median (black line); **(D)** in women with ER-positive tumors, this survival difference was lost.

A high value for the combined five-protein parameter was significantly associated with poor disease-free survival (*P* = 0.005, Figure 5A), whereas survival estimates calculated from the four-protein panel showed no significant difference between high and low values of the parameter (*P* = 0.076, Figure 5B). When women with ER-negative and ER-positive tumors were examined separately, the significant survival effect seen in the five-protein model was strengthened in the ER-negative group, in whom a high value for the combined parameter was significantly associated with poor disease-free survival (*P* = 0.003, Figure 5C). In contrast, in ER-positive patients the prognostic value of the combined parameter was no longer significant (*P* = 0.161, Figure 5D).

We also examined the association of the five-protein panel with clinical pathologic variables such as histological grade, tumor size, lymph node status and hormone receptor (ER and PR), and HER2 status, summarized in Table 1. Multiple linear regression was used to test the correlation between the individual serum protein levels (measured by SELDI-TOF MS) and the combined five-protein panel, and these clinicopathological characteristics (Table 3). Based on this analysis, the five-protein panel was significantly associated with tumor size (*P* = 0.018) and lymph node involvement (*P* = 0.016), further suggesting its potential to contribute to breast cancer prognosis.

**Table 3:** Multiple linear regression analysis of the association between five putative biomarker proteins and their combined value with clinical pathologic features

**Tumor variables**	***P *** **value**					
	**ApoCI**	**C3a-desArg**	**Transthyretin**	**ApoAI**	**ApoH fragment**	**Combined parameter**
Grade (G1, n = 22; G2, n = 64; G3, n = 96)	0.781	0.908	0.862	0.876	0.836	0.697
Tumor size (T ≤2 cm; T >2 cm)	0.314	0.867	0.004	0.921	0.516	0.018
LN (positive, n = 79; negative, n = 78)	0.182	0.503	0.397	0.119	0.499	0.016
ER (positive, n = 131; negative, n = 50)	0.550	0.756	0.151	0.517	0.124	0.341
PR (positive, n = 121; negative, n = 63)	0.723	0.774	0.010	0.171	0.340	0.825
HER2 (positive, n = 38; negative, n = 133)	0.509	0.280	0.968	0.018	0.733	0.087

ApoCI/AI/H, apolipoprotein CI/AI/H; C3a-desArg, C3a des-arginine anaphylatoxin; LN, lymph node involvement; ER, estrogen receptor; PR, progesterone receptor; HER2, human epidermal growth factor receptor 2.

## Discussion

Patient blood samples are an ideal source of disease biomarkers owing to their ease of access, and many studies have identified possible candidates, but few have overcome validation and reproducibility issues to achieve clinical application [22]. In the present study, we used protein chip mass spectrometry to discover and identify a unique panel of five serum proteins that, in combination, discriminate between sera from breast cancer patients and healthy volunteers with high sensitivity and specificity. The five-protein panel was developed by multivariate analysis of a larger group of proteins found to be significantly regulated in breast cancer, and validated on an independent data set. Whereas the sensitivity of the five-protein parameter was somewhat lower in the validation set than the training set, the specificity was slightly higher in the validation set. A simplified four-protein panel, from which data for a fragment of ApoH (*m/z* 3808) was omitted, showed considerably less specificity than the five-protein panel on both data sets (that is, it classified an increased number of false positives), but remained highly sensitive in detecting samples from women with BC.

When tested for its ability to predict disease-free patient survival, the median value of the five-protein parameter separated patients into significantly different groups, those with values above the median showing more rapid disease recurrence than those with values below the median. Using the four-protein parameter this significant discrimination was lost. Interestingly, the prognostic value of the five-protein parameter appeared to be restricted to women with ER-negative tumors, none of whom showed disease recurrence over the monitoring period if their five-protein parameter had a value below the median. Conversely, in this ER-negative group, almost half of the women had disease recurrence within the monitoring period if their five-protein parameter was above the median value. These distinctions were not seen among women with ER-positive tumors. Therefore we conclude that, in women with ER-negative tumors, the five-protein parameter appears to have strong prognostic value within the first five years. It is recognized that, owing to the use of endocrine therapy, ER-positive disease is less likely to relapse early [23]; therefore longer follow-up will be required to ascertain prognostic utility in this subgroup.

Using a combination of mass spectrometry and immunological methods, the proteins were identified as a fragment of ApoH, ApoCI, C3a-desArg, TTR, and ApoAI. Among this five-peak panel, three (C3a-desArg, TTR and ApoH) were increased in sera of the breast cancer patients compared to that of HV subjects, while ApoCI and ApoAI were decreased in cancer. Each of these serum proteins has previously been associated with breast cancer in various studies, but this study is the first to identify the unique prognostic value of combining their serum concentrations into a single parameter. The combined value was also significantly associated with tumor size (*P* = 0.018) and lymph node involvement (*P* = 0.016).

Human complement C3 is the most abundant complement protein in human serum. C3 convertase exists in two forms (C3bBb and C4bC2a) and cleaves only C3, a central molecule of the complement system, between residues 726 to 727 (Arg-Ser), generating C3b and an N-terminal fragment, C3a, (8.9 kDa) [24]. C3a has high biological activity and is able to trigger the degranulation of mast cells and basophils, which produces a local inflammatory response. The desArg form represents a stable inactivated form of complement C3a. C3a-desArg was previously observed to be higher in BC sera compared to healthy controls in several studies [14,21,25,26] with a *m/z* range of 8900 to 8941 observed on IMAC-Ni protein chips. Increased C3a-desArg serum levels have also been reported in hepatocellular and colorectal cancer [27,28]. In our study, we identified this protein at *m/z* 8916 on Q10 chips alone, with significant discrimination between breast cancer patients and healthy controls.

Transthyretin (TTR, also known as prealbumin) is a liver-derived secreted protein and is the major serum carrier of thyroid hormones, thyroxine and tri-iodothyronine. TTR is also involved in the transport of retinol through its interaction with retinol-binding proteins. Differential levels of TTR in serum have been linked to several cancers, including breast [29,30], ovarian [31] and hepatocellular carcinomas [32]. Five isoforms of TTR have been previously demonstrated by MALDI analysis after immunoaffinity capture [33]: full-length TTR (13,758 Da), a form truncated N-terminally by 10 residues (12,210 Da), and the three modified isoforms (Cys-TTR at *m/z* 13876, CysGly-TTR at *m/z* 13924, and glutathionylated-TTR at *m/z* 14062). In our study, we identified the peak at *m/z* 13870 as full-length TTR; however, a peak at *m/z* 13756 detected by Q10 protein chip was also significantly upregulated in the serum of breast cancer patients (Table S1 in Additional file 2). Only the isoform that most likely corresponds to Cys-TTR (*m/z* 13870 in this study) was computationally selected into the final five-protein panel.

Apolipoproteins bind lipids to form lipoproteins that transport the lipids through the lymphatic and circulatory systems. Serum and plasma lipoprotein metabolism is regulated and controlled by the specific apolipoprotein (Apo-) constituents of the various lipoprotein classes such as ApoAI, ApoCI, ApoH (beta2 glycoprotein) and others. Several classes of apolipoprotein in serum or plasma have been discovered as putative breast cancer biomarkers using proteomic techniques including SELDI-TOF, MALDI-TOF/TOF, 2D-iTRAQ-LC-MS/MS, and 2D-LC MS/MS [19-21,30,34]. We observed that levels of ApoAI and ApoCI were significantly downregulated in breast cancer patients, while a peptide identified as a fragment of ApoH was significantly higher in BC. A previous study also identified both ApoAI and ApoCI by SELDI-TOF as part of a multiprotein panel evaluated as a predictor of metastatic relapse in high-risk BC patients [20]. Decreased serum ApoAI has also been found in other types of cancer including ovarian [31] and bladder carcinomas [30]. ApoAI and ApoAI mimetic peptides have been shown to inhibit tumor development in a mouse model of ovarian cancer, suggesting that ApoAI may not only have potential as a biomarker, but may also have therapeutic utility in this disease [35]. Serum ApoCI has also been previously found to be decreased in breast cancer patients compared to healthy control groups [21]. ApoH or beta2 glycoprotein was recognized immunologically over 30 years ago as being increased in the serum of breast cancer patients [36], but the 3808 Da ApoH fragment that we found to be increased in breast cancer sera has not been reported previously.

## Conclusions

While many serum proteins have been found to differ significantly in concentration between healthy subjects and those with breast cancer, they have little discriminatory or prognostic value when used as single markers. In contrast, this study has shown that patients with greater than median values of a combined biomarker calculated from the concentrations of five serum proteins have significantly shorter disease-free survival times than those with below-median values of this parameter. Notably, the prognostic value of this five-protein parameter appeared to be greatest in women with ER-negative tumors. Therefore in this patient group the combined biomarker may show clinical utility as an adjunct to other pathological variables in predicting patient outcome. This needs to be confirmed in larger patient cohorts, and the development of the new marker panel in an immunoassay format (for example, a multiplexed ELISA) will facilitate its further evaluation as a novel tool in the management of patients with breast cancer.

## Abbreviations

ACN: Acetonitrile; Apo: Apolipoprotein; AUC: Area under the curve; BC: Breast cancer patient; C3a-desArg: C3a des-arginine anaphylatoxin; ER: Estrogen receptor; HER2: Human epidermal growth factor receptor 2; HV: Healthy volunteer; Ig: Immunoglobulin; MALDI-TOF: Matrix-assisted laser desorption/ionization time-of-flight; MS: Mass spectrometry; PBS: Phosphate-buffered saline; PR: Progesterone receptor; ROC: Receiver operating characteristic; RP-HPLC: Reverse-phase high-performance liquid chromatography; SELDI-TOF: Surface-enhanced laser desorption/ionization time-of-flight; TFA: Trifluoroacetic acid; TTR: Transthyretin.

## Competing interests

All authors declare that they have no competing interests.

## Authors’ contributions

LC collected the MS data and performed the statistical analysis, contributed substantially to data interpretation, and drafted the first manuscript. KM contributed to study design, acquisition of data on patient outcomes, and data interpretation. LP contributed to acquisition of data for protein biomarker identification. FMB and DJM contributed to study design and data interpretation. RCB coordinated the study design, data interpretation, and manuscript preparation and revision. All authors read and approved the final manuscript.

## Supplementary Material

Additional file 1: Figure S1 Statistical analysis of the protein peaks detected on H50, IMAC30, CM10 and Q10 protein chip arrays. Click here for file

Additional file 2: Table S1 Significant peaks and receiver operating charactistic-area under the curve (ROC-AUC) observed between breast cancer patients (BC) and healthy volunteers (HV) across four chip types. Click here for file

Additional file 3: Table S2 Peptide sequences identified by matrix-assisted laser desorption/ionization time-of-flight/time-of-flight mass spectrometry (MALDI-TOF/TOF MS). Click here for file
