# Supplementary material for: Novel serum protein biomarker panel revealed by mass spectrometry and its prognostic value in breast cancer
Source: Breast Cancer Res. 2014 Jun 16;16(3):R63. doi: 10.1186/bcr3676 (PMC4095593; doi:10.1186/bcr3676)
Supplement: Additional file 1: Figure S1 — Statistical analysis of the protein peaks detected on H50, IMAC30, CM10 and Q10 protein chip arrays. [file bcr3676-S1.pdf]

Supplementary Table S2
Peptide sequences identified by MALDI-TOF/TOF MS

Protein identity	Accession	Theoretical pI/MW	Peptide mass	Peptide sequence by MALDI-TOF-TOF
ApoAI	P02647	5.27/28078.62	*m/z* 704.3279	K.ETEGLR.Q
			m/z 732.3503	K.DLEEVK.A
			m/z 831.3713	R.LAEYHAK.A
			m/z 869.4779	R.QKVEPLR.A
			m/z 873.4126	R.AELQEGAR.Q
			m/z 896.4201	K.LHELQEK.L
			m/z 1008.5228	R.ARAHVDALR.T
			m/z 1012.5083	K.AKPALEDLR.Q
			m/z 1031.4388	K.LSPLGEEMR.D
			m/z 1152.5613	R.QKLHELQEK.L
			m/z 1157.5508	R.LEALKENGGAR.L
			m/z 1215.5619	K.ATEHLSTLSEK.A
			m/z 1252.5560	K.VQPYLDDFQK.K
			m/z 1299.5087	K.WQEEMELYR.Q
			m/z 1301.5539	R.THLAPYSDELR.Q
			m/z 1307.5261	K.ETEGLRQEMSK.D
			m/z 1318.5744	K.LSPLGEEMRDR.A
			m/z 1323.5383	K.ETEGLRQEMSK.D
			m/z 1380.6466	K.VQPYLDDFQKK.W
			m/z 1400.6022	R.DYVSQFEGSALGK.Q
			m/z 1427.6592	K.KWQEEMELYR.Q
			m/z 1451.6726	K.AKVQPYLDDFQK.K
			m/z 1462.7777	R.VKDLATVYVDVLK.D
			m/z 1467.7623	K.VEPLRAELQEGAR.Q
			m/z 1585.7484	R.THLAPYSDELRQR.L
			m/z 1815.7964	K.DSGRDYVSQFEGSALGK.Q
			m/z 1881.8834	K.LLDNWDSVTSTFSKLR.E
			m/z 1908.9398	K.LHELQEKLSPLGEEMR.D
			m/z 2064.0144	R.AHVDALRTHLAPYSDELR.Q
			m/z 2202.2336	K.LREQLGPVTQEFWDNLEK.E
			m/z 2209.0707	K.QLNLKLLDNWDSVTSTFSK.L
			m/z 2224.3566	K.AKPALEDLRQGLLPVLESFK.V
			m/z 2598.3533	R.QGLLPVLESFKVSFLSALEEYTK.K
Transthyretin	P02766	5.31/13761.41	*m/z* 1366.7393	R.GSPAINNVAVHVFR.K
			m/z 2451.1833	K.ALGISPFHEHAEVVFTANDSGPR.R
			m/z 2645.2229	R.RYTIAALLSPYSTTAVVTNPK.E
APOH	P02749	8.34/38298.16	*m/z* 1445.6384	R.VCPFAGGILENGGAVR.Y
