# Supplementary material for: Novel serum protein biomarker panel revealed by mass spectrometry and its prognostic value in breast cancer
Source: Breast Cancer Res. 2014 Jun 16;16(3):R63. doi: 10.1186/bcr3676 (PMC4095593; doi:10.1186/bcr3676)
Supplement: Additional file 2: Table S1 — Significant peaks and receiver operating charactistic-area under the curve (ROC-AUC) observed between breast cancer patients (BC) and healthy volunteers (HV) across four chip types. [file bcr3676-S2.pdf]

Supplementary Figure S1

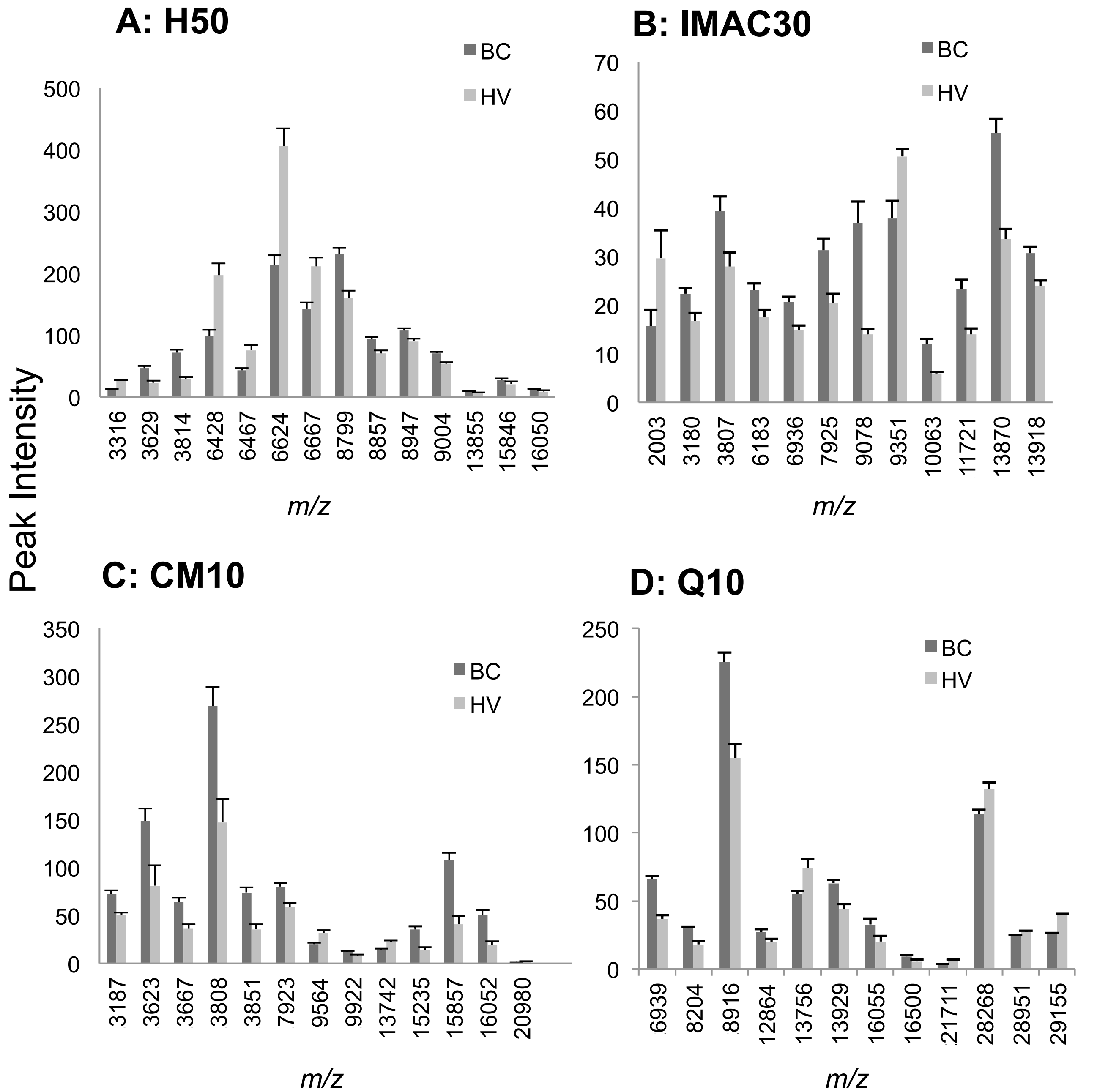

Supplementary Figure S1. Comparison of discriminatory protein peaks detected on H50, IMAC30, CM10 and Q10 protein chip arrays. Determined by univariate analysis, 14 peaks from H50 (A), 12 from IMAC30 (B), 13 from CM10 (C), and 12 from Q10 (D) were significantly either up- or down- regulated ($p<0.005$) in sera from BC patients compared to HV patients.
