# Supplementary material for: Novel serum protein biomarker panel revealed by mass spectrometry and its prognostic value in breast cancer
Source: Breast Cancer Res. 2014 Jun 16;16(3):R63. doi: 10.1186/bcr3676 (PMC4095593; doi:10.1186/bcr3676)
Supplement: Additional file 3: Table S2 — Peptide sequences identified by matrix-assisted laser desorption/ionization time-of-flight/time-of-flight mass spectrometry (MALDI-TOF/TOF MS). [file bcr3676-S3.pdf]

Supplementary Table S1

Significant peaks and ROC-AUC observed between BC and HV samples in the training set across four chip types

m/z	P value				ROC-AUC	
	H50	IMAC	CM10	Q10	Up-regulated	Down-regulated
2003		0.001				0.305
3072				0.000	0.753	
3180		0.005			0.67	
3187			0.000		0.703	
3316	0.000					0.205
3623			0.000		0.753	
3629	0.000				0.775	
3667			0.000		0.718	
3807		0.001			0.646	
**3808**			**0.000**		**0.749**	
3814	0.000				0.838	
3851			0.000		0.777	
6183		0.003			0.624	
6428	0.000					0.273
6467	0.000					0.294
**6624**	**0.000**					**0.232**
6667	0.001					0.323
6936		0.000			0.729	
6939				0.001		0.262
7923			0.001		0.667	
7925		0.004			0.671	
8204				0.005	0.695	
8799	0.000				0.721	
8857	0.000				0.699	
**8916**				**0.001**	**0.755**	
8947	0.005				0.642	
9004	0.000				0.721	
9078		0.000			0.713	
9351		0.001				0.347
9423			0.006			0.357
9564			0.000			0.31
9922			0.000		0.684	
10063		0.001			0.696	
11721		0.004			0.673	
12864				0.002		0.378
13742			0.001			
13756				0.000	0.695	
13855	0.000				0.679	
**13870**		**0.000**			**0.788**	
13918		0.004			0.674	
13929				0.000		0.342

15235		0.000		0.775	
15846	0.001			0.663	
15857		0.000		0.817	
16050	0.002			0.661	
16052		0.000		0.813	
16055			0.000	0.655	
16500			0.000	0.608	
20980		0.000			0.276
21711			0.001		0.319
**28268**			**0.001**		**0.295**
28951			0.003		0.335
29155			0.004		0.323

The five peaks selected for analysis in this study are indicated in bold type.
